# Mechanism for insulin-like peptide 5 distinguishing the homologous relaxin family peptide receptor 3 and 4

**DOI:** 10.1038/srep29648

**Published:** 2016-07-11

**Authors:** Meng-Jun Hu, Xiao-Xia Shao, Jia-Hui Wang, Dian Wei, Yu-Qi Guo, Ya-Li Liu, Zeng-Guang Xu, Zhan-Yun Guo

**Affiliations:** 1 Research Centre for Translational Medicine at East Hospital, College of Life Sciences and Technology, Tongji University, Shanghai, China

## Abstract

The relaxin family peptides play a variety of biological functions by activating four G protein-coupled receptors, RXFP1–4. Among them, insulin-like peptide 5 (INSL5) and relaxin-3 share the highest sequence homology, but they have distinct receptor preference: INSL5 can activate RXFP4 only, while relaxin-3 can activate RXFP3, RXFP4, and RXFP1. Previous studies suggest that the A-chain is responsible for their different selectivity for RXFP1. However, the mechanism by which INSL5 distinguishes the homologous RXFP4 and RXFP3 remains unknown. In the present work, we chemically evolved INSL5 *in vitro* to a strong agonist of both RXFP4 and RXFP3 through replacement of its five B-chain residues with the corresponding residues of relaxin-3. We identified four determinants (B2Glu, B9Leu, B17Tyr, and a rigid B-chain C-terminus) on INSL5 that are responsible for its inactivity at RXFP3. In reverse experiments, we grafted these determinants onto a chimeric R3/I5 peptide, which contains the B-chain of relaxin-3 and the A-chain of INSL5, and retains full activation potency at RXFP3 and RXFP4. All resultant R3/I5 mutants retained high activation potency towards RXFP4, but most displayed significantly decreased or even abolished activation potency towards RXFP3, confirming the role of these four INSL5 determinants in distinguishing RXFP4 from RXFP3.

The relaxin family is a group of peptide hormones, including relaxin (primates express two relaxins, namely relaxin-1 and relaxin-2), relaxin-3 (also known as INSL7), and insulin-like peptides 3–6 (INSL3, INSL4, INSL5, and INSL6)^15^. The relaxin family is a branch of the insulin superfamily that also includes insulin and insulin-like growth factor 1 and 2 (IGF-1 and IGF-2). The mature relaxin family peptides are typically composed of two polypeptide chains with three disulfide bonds and play a variety of biological functions^1^,^2^,^3^,^4^,^5^, such as regulation of reproduction, food intake, stress response, and glucose homeostasis. So far, four G protein-coupled receptors have been identified as the relaxin family peptide receptors, namely RXFP1–4. Relaxin and INSL3 are the cognate agonists of the homologous RXFP1 and RXFP2, respectively^6^,^7^. Relaxin-3 and INSL5 are the cognate agonists of the homologous RXFP3 and RXFP4, respectively^8^,^9^. In addition, relaxin-3 has been shown to activate RXFP1 and RXFP4 *in vitro* with high efficiency^10^,^11^.

Among the four known relaxin family peptide receptors, the homologous RXFP1 and RXFP2 belong to the leucine-rich repeat (LRR)-containing G protein-coupled receptor subfamily. Both RXFP1 and RXFP2 contain a large extracellular N-terminal domain containing 10 LRRs and a unique N-terminal low-density lipoprotein receptor type A (LDLa) module. The LRR module forms the high affinity ligand-binding site that primarily interacts with the essential B-chain residues of their ligands^12^,^13^,^14^, and the LDLa module is critical for receptor activation^15^,^16^,^17^,^18^. The extracellular loops of RXFP1 and RXFP2 form a low affinity ligand-binding site that primarily interacts with the A-chain residues of their ligands^19^,^20^,^21^,^22^,^23^. On the other hand, the homologous RXFP3 and RXFP4 are classical peptide receptors, with a short N-terminal domain, and thus, their extracellular loops form the primary ligand-binding site. Indeed, recent studies suggest that a negatively charged ExxxD motif at the extracellular end of the second transmembrane domain of RXFP3 and RXFP4 plays a critical role in ligand binding through interaction with the positively charged B-chain Arg residues of their ligands^24^,^25^,^26^,^27^.

Among the relaxin family peptides, INSL5 and relaxin-3 share the highest sequence homology and similar three-dimensional structures^28^,^29^. However, they have distinct receptor preferences. INSL5 can activate RXFP4 only; although it can also bind RXFP3 with a lower affinity, it cannot activate the receptor. In contrast, relaxin-3 can activate RXFP3, RXFP4, and RXFP1. So far, the mechanism of their distinct receptor selectivity is not fully understood. Previous studies showed that the chimeric R3/I5 peptide, containing the B-chain of relaxin-3 and the A-chain of INSL5, loses activation potency towards RXFP1, but retains high activation potency towards both RXFP3 and RXFP4^30^,^31^. Thus, the A-chain of INSL5 and relaxin-3 determines their different selectivity against RXFP1. However, the mechanism of their distinct selectivity against RXFP3 is remained unknown. As shown in Fig. 1A, their B-chains contain residues conserved in both INSL5 and relaxin-3, such as B13Arg, B23Arg, B16Ile, and B24Trp (numbered according to INSL5), that are important for binding and activation of RXFP4 and/or RXFP3^32^,^33^,^34^. Moreover, a free acidic B-chain C-terminus is also important for the activity of INSL5 and relaxin-3^35^,^36^,^37^. On the other hand, INSL5 and relaxin-3 also contain unique residues in their B-chains (Fig. 1A), and some of these residues are likely responsible for their distinct selectivity against RXFP3.

In the present work, we first replaced some exposed INSL5 B-chain residues with the corresponding residues of relaxin-3 (Fig. 1A,C, shown in red), in order to identify the key residues important for INSL5 selectivity towards RXFP4 over RXFP3. Our results disclosed four determinants responsible for inactivity of INSL5 against RXFP3. When the four determinants were all replaced by the corresponding residues of relaxin-3, the resultant INSL5 mutant acquired high activation potency towards both RXFP4 and RXFP3. Second, we conducted reverse experiments, in which these determinants were grafted, either individually or combined, onto the chimeric R3/I5 peptide that efficiently activates both RXFP3 and RXFP4^30^. All resultant R3/I5 mutants retained high activation potency towards RXFP4, while most displayed significantly decreased or even abolished activation potency towards RXFP3, confirming the role of these four INSL5 determinants in receptor selectivity. Our present study discloses the mechanism by which INSL5 distinguishes RXFP4 from the homologous RXFP3, and sheds new light on how the relaxin family peptides interact with their receptors.

## Results

### Preparation and characterization of INSL5 and R3/I5 mutants

All INSL5 and R3/I5 mutants were overexpressed in *Escherichia coli* as single-chain precursors and formed inclusion bodies. To improve solubilisation of the precursors from inclusion bodies, in the present work we used an *S*-sulfonation approach, by which inter- and intra-molecular disulfide bonds were broken and the sulfhydryl moieties of Cys residues were reversibly modified by negatively charged sulfonate moieties. After purification by metal ion affinity chromatography, the S-sulfonated precursors were subjected to *in vitro* refolding using a reduction-oxidation procedure that has been used for refolding the wild-type INSL5 precursor and R3/I5 precursor^38^,^39^. Except for the precursor of [L(B9)R,Y(B17)F]INSL5, all other precursors could be refolded with considerable yields using this approach. For the precursor of [L(B9)R,Y(B17)F]INSL5, only the solubilized precursor without *S*-sulfonation could be refolded with a considerable yield using a disulfide-reshuffling approach^40^. This folding behaviour has been observed in the refolding of relaxin-3 precursors^26^,^40^,^41^. All of the folded precursors were purified by high performance liquid chromatography (HPLC) using a C18 reverse-phase column and confirmed by mass spectrometry. Their measured molecular masses were consistent with the theoretical values (Table 1), confirming presence of the expected mutation in these INSL5 and R3/I5 mutants.

Thereafter, the purified folded single-chain precursors were converted to their mature two-chain forms by enzymatic treatment and purified by reverse-phase HPLC according to our previous procedures^38^,^39^. As analysed by mass spectrometry, all mature INSL5 and R3/I5 mutants showed the expected molecular mass (Table 1), confirming correct *in vitro* processing of these precursors. Purity of these mature INSL5 and R3/I5 mutants was analysed by HPLC using an analytical C18 reverse-phase column (Fig. 2A,C). All mature mutants showed a single symmetrical peak, suggesting they were homogeneous. The secondary structures of these mature INSL5 and R3/I5 mutants were analysed by circular dichroism (Fig. 2B,D). The spectra of the mutants were similar to that of wild-type INSL5 or wild-type R3/I5, suggesting that these mutations did not disturb the overall structure of INSL5 and R3/I5.

### A flexible B-chain C-terminus is required for activation of RXFP3

**Figure 1:**
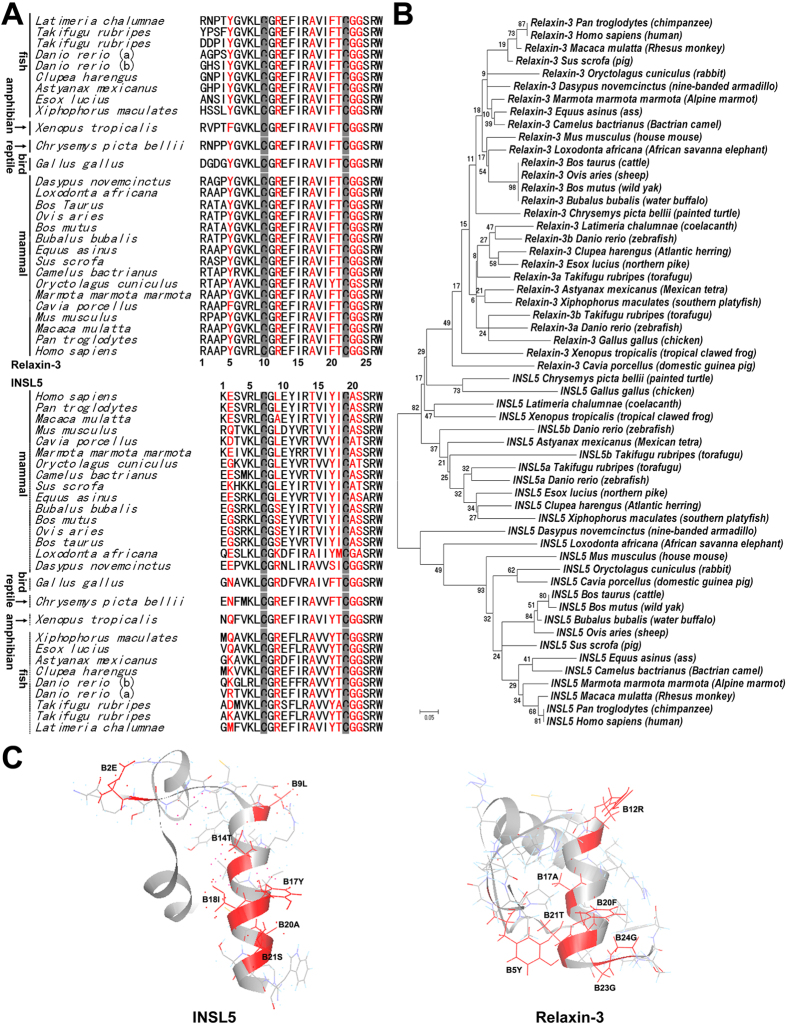
Sequence alignment and structure comparison of INSL5 and relaxin-3. (**A**) Amino acid sequence alignment of the INSL5 and relaxin-3 B-chains from different species. The residues exchanged from relaxin-3 to INSL5 in the present work are shown in red. (**B**) Phylogenetic relationship of relaxin-3s and INSL5s across vertebrates based on their full-length amino acid sequences, which were obtained from the NCBI website (http://www.ncbi.nlm.nih.gov/gene) and analyzed using the software MEGA. (**C**) The previously reported solution structures of INSL5 and relaxin-3. The residues exchanged from relaxin-3 to INSL5 are shown as red sticks and labelled.

**Figure 2:**
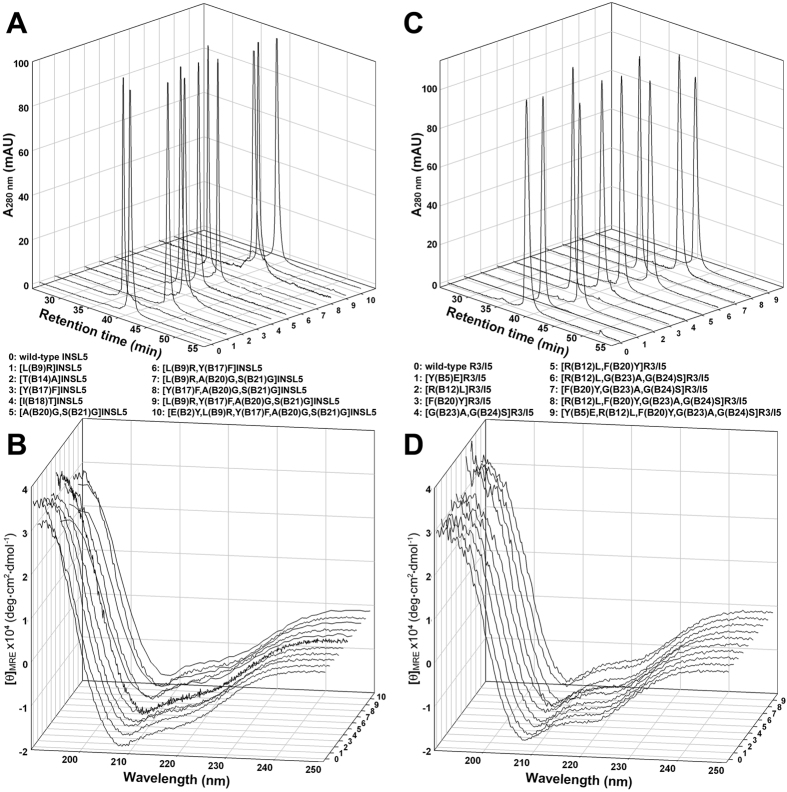
Characterisation of the mature INSL5 and R3/I5 mutants. (**A**) Purity analysis of INSL5 mutants using C18 reverse-phase HPLC. (**B**) Structural analysis of INSL5 mutants using circular dichroism. (**C**) Purity analysis of R3/I5 mutants using C18 reverse-phase HPLC. (**D**) Structural analysis of R3/I5 mutants using circular dichroism.

INSL5 and relaxin-3 have distinct conformations at their B-chain C-terminus, although their overall structures are quite similar (Fig. 1C). The B-chain C-terminus of relaxin-3 and the chimeric R3/I5 forms a folded-back conformation^28^,^31^, while that of INSL5 forms an extended α-helical conformation^29^. This difference is probably caused by the different residues at their B-chain C-terminus (Fig. 1A). The B23–B24 positions of relaxin-3s from different species are always occupied by two small Gly residues, which have the highest degree of freedom. In contrast, the corresponding positions (B20–B21) of INSL5s are typically occupied by larger Ala and Ser residues.

**Figure 3:**
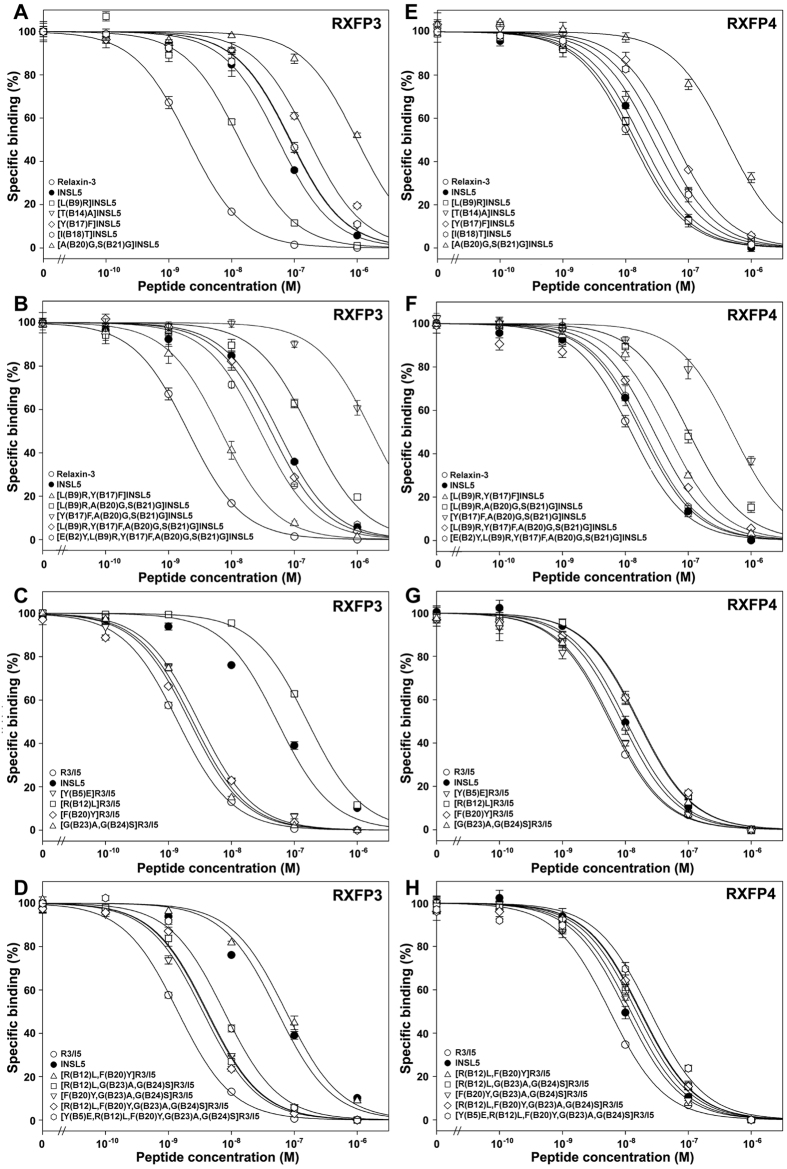
Receptor binding activities of the mature INSL5 and R3/I5 mutants towards RXFP3 and RXFP4. A NanoLuc-conjugated R3/I5 peptide was used as tracer and HEK293T cells transiently overexpressing human RXFP3 or human RXFP4 were used as receptor source. The tracer concentration used in these assays was 0.5 nM. The nonspecific binding was obtained by competition with 1.0 μM of relaxin-3. The measured data were expressed as mean ± SE (*n* = 3) and fitted to sigmoidal curves using the software SigmaPlot 10.0. The calculated pIC_50_ values are summarized in Table 2.

**Figure 4:**
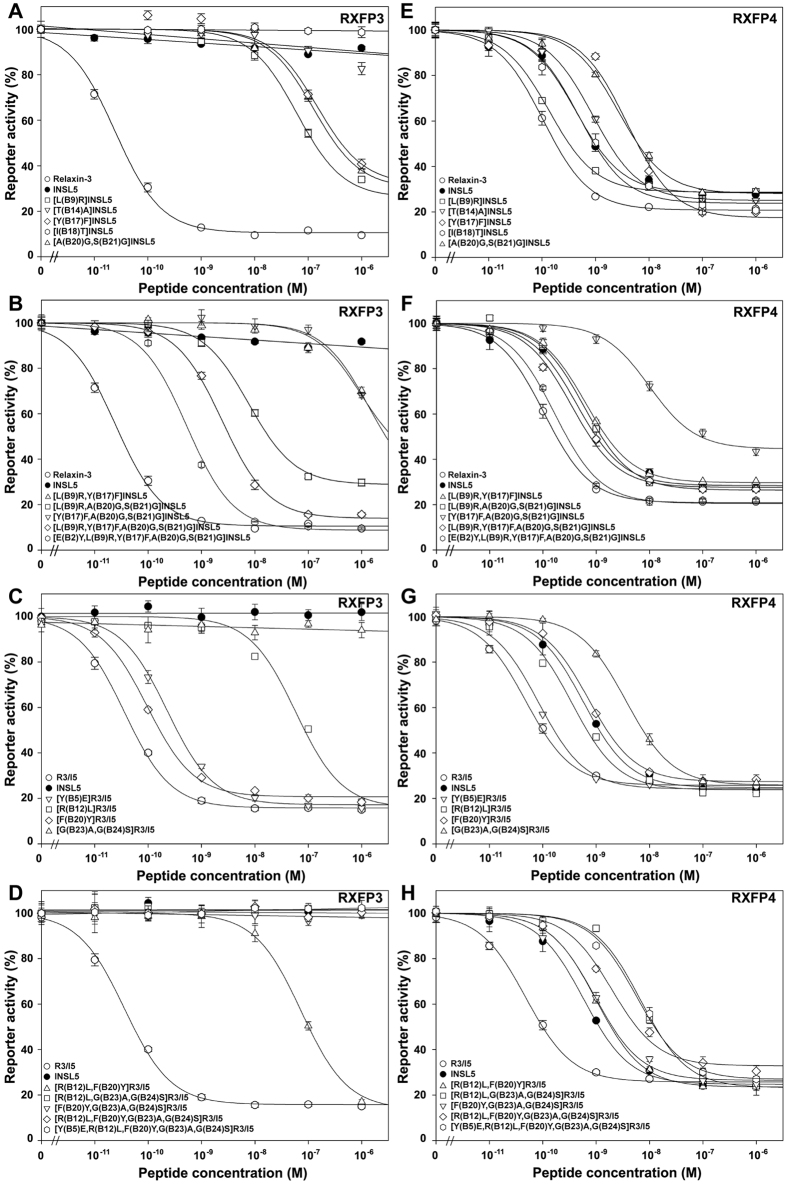
Receptor activation activities of the mature INSL5 and R3/I5 mutants towards RXFP3 and RXFP4. HEK293T cells transiently cotransfected by human RXFP3 or human RXFP4 and a CRE-controlled NanoLuc reporter were used for the activation assays. The measured data were expressed as mean ± SE (*n* = 3) and fitted to sigmoidal or linear curves using the software SigmaPlot 10.0. The calculated pEC_50_ values are summarized in Table 2.

In our previous work^24^, when the B20–B21 residues of human INSL5 were replaced by two Gly residues, the resultant [A(B20)G,S(B21)G]INSL5 showed ~23-fold lower binding potency and ~6-fold lower activation potency towards RXFP4, compared with wild-type INSL5 (Figs 3E and 4E and Table 2). Thus, a rigid B-chain C-terminus is required for INSL5 to activate RXFP4. When [A(B20)G,S(B21)G]INSL5 was tested on RXFP3, its binding potency was also ~18-fold lower than that of wild-type INSL5 (Fig. 3A and Table 2). However, it could activate RXFP3, although its efficiency was much lower (~5000-fold) than that of relaxin-3, the cognate agonist of RXFP3 (Fig. 4A and Table 2). In contrast, wild-type INSL5 had no detectable activation potency towards RXFP3 (Fig. 4A and Table 2), consistent with previous studies^9^,^42^, although it could bind RXFP3 with a lower affinity (~27-fold lower than that of relaxin-3). Thus, a flexible B-chain C-terminus is required for INSL5 to activate RXFP3.

To confirm the role of the B-chain C-terminal conformation in distinguishing RXFP4 from RXFP3, we conducted a reverse experiment, in which a rigid B-chain C-terminus was introduced into the chimeric R3/I5 peptide. The resultant [G(B23)A,G(B24)S]R3/I5 retained high binding potencies towards both RXFP3 and RXFP4 compared with wild-type R3/I5 (Fig. 3C,G and Table 2), suggesting that the B-chain C-terminal conformation of R3/I5 is not relevant for binding these receptors. However, this R3/I5 mutant completely lost activation potency towards RXFP3 (Fig. 4C and Table 2), suggesting that a flexible B-chain C-terminus is essential for R3/I5 to activate RXFP3. On the other hand, this R3/I5 mutant could activate RXFP4, although its activation potency was ~76-fold lower than that of wild-type R3/I5 and ~8-fold lower than that of INSL5 (Fig. 4G and Table 2). Thus, RXFP4 is tolerant to a rigid B-chain C-terminus for R3/I5.

In summary, RXFP3 and RXFP4 have different requirements for the B-chain C-terminal conformation of ligands for efficient activation. RXFP4 is largely tolerant to a flexible or a rigid B-chain C-terminus, although it somewhat prefers a rigid B-chain C-terminus for INSL5 and a flexible B-chain C-terminus for R3/I5. In contrast, RXFP3 stringently requires a flexible B-chain C-terminus for both ligands.

### A positively charged Arg at the B9 position is required for activation of RXFP3

The B9 residue of INSL5s from different species is variable (Fig. 1A), while the corresponding position (B12) of relaxin-3 is always occupied by an Arg residue that has been shown to be involved in binding and activation of RXFP3. After an Arg residue was introduced to the B9 position of human INSL5, the resultant [L(B9)R]INSL5 showed slightly higher binding and activation potencies towards RXFP4 (Figs 3E and 4E and Table 2). Thus, introduction of an Arg residue at the B9 position of INSL5 has slight beneficial effects on binding and activation of RXFP4. When tested on RXFP3, [L(B9)R]INSL5 showed ~4-fold higher binding potency than wild-type INSL5 (Fig. 3A and Table 2). More importantly, this mutant acquired low but significant activation potency towards RXFP3 (Fig. 4A and Table 2). Thus, a positively charged Arg at the B9 position is required for INSL5 to activate RXFP3.

To confirm the role of the B-chain Arg residue in distinguishing RXFP4 from RXFP3, we carried out a reverse experiment in which B12Arg of R3/I5 was replaced by the corresponding B9Leu residue of INSL5. Towards RXFP3, the resultant [R(B12)L]R3/I5 showed ~110-fold lower binding potency and ~1600-fold lower activation potency compared with wild-type R3/I5 (Figs 3C and 4C and Table 2), suggesting that B12Arg is critical for R3/I5 to bind and activate RXFP3. However, this mutant R3/I5 showed only slightly lower binding and activation potencies towards RXFP4 (Figs 3G and 4G and Table 2). Thus, B12Arg of R3/I5 has only a small beneficial effect on binding and activation of RXFP4.

In summary, RXFP3 and RXFP4 have different requirements for the B9 position of INSL5 and the corresponding B12 position of R3/I5 for efficient activation. RXFP4 is largely tolerant to an Arg or a Leu residue, although it slightly prefers an Arg residue for both R3/I5 and INSL5. However, RXFP3 strongly requires an Arg residue for efficient activation.

### A hydrophobic Phe at the B17 position is required for activation of RXFP3

The B17 position of INSL5s from different species is always occupied by a Tyr residue, while the corresponding position (B20) of relaxin-3 is typically occupied by a Phe residue (Fig. 1A). When a Phe residue was introduced to the B17 position of human INSL5, the resultant [Y(B17)F]INSL5 showed ~3-fold lower binding potency and ~9-fold lower activation potency towards RXFP4 (Figs 3E and 4E and Table 2). Thus, RXFP4 prefers a Tyr residue at the B17 position of INSL5 for both binding and activation. When tested on RXFP3, [Y(B17)F]INSL5 also showed ~3-fold lower binding potency compared with wild-type INSL5 (Fig. 3A and Table 2). However, this INSL5 mutant could activate RXFP3 with a low efficiency (Fig. 4A and Table 2). Thus, a hydrophobic Phe residue at the B17 position is required for INSL5 to activate RXFP3.

In a reverse experiment, when B20Phe of R3/I5 was replaced by the corresponding B17Tyr residue of INSL5, the resultant [F(B20)Y]R3/I5 retained almost normal binding potency and slightly lower (~3-fold) activation potency towards RXFP3 (Figs 3C and 4C and Table 2). Towards RXFP4, this R3/I5 mutant had a slightly lower (~3-fold) binding potency and moderately lower (~15-fold) activation potency (Figs 3G and 4G and Table 2). Thus, both RXFP3 and RXFP4 have a slight preference for a Phe residue at the B20 position of R3/I5.

In summary, RXFP3 and RXFP4 have a different preference for the B17 position of INSL5 and the corresponding B20 position of R3/I5. RXFP4 prefers a Tyr residue at the B17 position for INSL5, but prefers a Phe residue at the corresponding B20 position for R3/I5. RXFP3 has a slight preference for a Tyr residue at the B20 position for R3/I5, but requires a Phe residue at the corresponding B17 position of INSL5 for activation.

### Replacement of B14 or B18 residue of INSL5 has no significant effects on RXFP3 activation

The B14 and B18 positions of INSL5s from difference species are typically occupied by Thr and Ile, respectively, while the corresponding positions (B17 and B21) of relaxin-3 are typically occupied by Ala and Thr (Fig. 1A). After the B14 or B18 position of human INSL5 was replaced by the corresponding residue of human relaxin-3, the resultant [T(B14)A]INSL5 and [I(B18)T]INSL5 showed almost normal binding and activation potencies towards RXFP4 (Figs 3E and 4E and Table 2). Towards RXFP3, both mutants retained binding potency similar to that of wild-type INSL5 (Fig. 3A and Table 2), and had no detectable activation potency (Fig. 4A and Table 2). We deduced that the B14 and B18 residues of INSL5 are not involved in binding and activation of RXFP3 and RXFP4, and thus, we did not do reverse experiments on R3/I5 at these positions.

### Combining mutations improves activation potency of INSL5 towards RXFP3

Our work identified three positions (B20Ala–B21Ser, B9Leu, and B17Tyr) that are responsible for the receptor selectivity of INSL5. When these positions were replaced individually by the corresponding residues of relaxin-3, the resultant INSL5 mutants acquired low but significant activation potency towards RXFP3. To generate INSL5 mutants with higher activation potency towards RXFP3, we attempted to combine these mutations. When mutations were introduced to both the B9 and B17 positions, the resultant [L(B9)R,Y(B17)F]INSL5 showed a slightly lower (~3-fold) binding potency and nearly normal activation potency towards RXFP4 (Figs 3F and 4F and Table 2), compared with wild-type INSL5. Thus, the adverse effect of the Y(B17)F mutation on RXFP4 activation was compensated by the beneficial effect of the L(B9)R mutation. On the other hand, this combination had a significant beneficial effect on binding potency towards RXFP3 (Fig. 3B and Table 2). Unfortunately, this combination had an adverse effect on activation potency towards RXFP3: the mutant showed ~10-fold lower activation potency than its parent mutants (Fig. 4B and Table 2). Thus, combining the L(B9)R and Y(B17)F mutations does not improve activation potency of INSL5 towards RXFP3, although it improves binding potency towards the receptor.

When mutations were introduced to both the B17 and B-chain C-terminal positions, the resultant [Y(B17)F,A(B20)G,S(B21)G]INSL5 showed lower binding and activation potencies towards RXFP4, compared with its parent mutants (Figs 3F and 4F and Table 2), likely due to accumulation of the adverse effects of both mutations. This combination also had an adverse effect on binding and activation of RXFP3: the mutant showed even lower binding and activation potencies compared with its parent mutants (Figs 3B and 4B and Table 2). Thus, combining Y(B17)F and [A(B20)G,S(B21)G] mutations has adverse effects on activation and binding of RXFP3 and RXFP4.

When mutations were introduced to the B9 and B-chain C-terminal positions, the resultant [L(B9)R,A(B20)G,S(B21)G]INSL5 showed a moderately decreased (~6-fold) binding potency and a nearly normal activation potency towards RXFP4, compared with wild-type INSL5 (Figs 3F and 4F and Table 2). This is likely due to the compensation of the adverse effect of the [A(B20)G,S(B21)G] mutation by the beneficial effect of the L(B9)R mutation. Towards RXFP3, the mutant acquired higher activation potency compared with its parent mutants, although its binding potency was not further improved (Figs 3B and 4B and Table 2). Thus, combining the L(B9)R and [A(B20)G,S(B21)G] mutations has beneficial effects on activation of RXFP3.

When mutations were introduced to all three positions, the resultant 4-mutation INSL5, [L(B9)R,Y(B17)F,A(B20)G,S(B21)G]INSL5, retained almost normal binding and activation potencies towards RXFP4 compared with wild-type INSL5 (Figs 3F and 4F and Table 2), a result of balance between the adverse effects and the beneficial effects of these mutations. At RXFP3, the 4-mutation INSL5 acquired significantly higher activation potency than its parent mutants (Fig. 4B and Table 2), although the binding potency was not significantly increased (Fig. 3B and Table 2). Thus, a combination of the four mutations in INSL5 has a significant beneficial effect on RXFP3 activation.

In reverse experiments, combination of these INSL5 determinants onto R3/I5 had no serious effects on binding and activation of RXFP4: all mutants showed similar binding and activation potencies compared with their parent mutants (Figs 3H and 4H and Table 2). At RXFP3, these R3/I5 mutants retain almost normal binding potencies compared with their parent mutants (Fig. 3D and Table 2). However, their activation potencies towards RXFP3 were either further decreased or completely undetectable (Fig. 4D and Table 2). Thus, these INSL5 determinants, especially B20Ala–B21Ser and B9Leu, play a critical role in distinguishing RXFP4 from RXFP3.

### A B-chain N-terminal mutation further increases activation potency of INSL5 towards RXFP3

As shown above, combination of the four mutations improved activation potency of INSL5 towards RXFP3. However, activation potency of the 4-mutation INSL5 towards RXFP3 was still ~100-fold lower than that of relaxin-3, the cognate agonist of RXFP3. We found that the B-chain N-terminal residues of relaxin-3 and INSL5 were quite different (Fig. 1A). For example, the B5 position of relaxin-3 from different species is always occupied by a Tyr residue, while the corresponding B2 position of INSL5 is occupied by a Glu residue in most cases. When a Tyr residue was introduced to the B2 position of the 4-mutation INSL5, the resultant 5-mutation INSL5, [E(B2)Y,L(B9)R,Y(B17)F,A(B20)G,S(B21)G]INSL5, showed slightly higher binding and activation potencies towards RXFP4 compared with the parent mutant (Figs 3F and 4F and Table 2), suggesting that a Tyr residue at B2 position had a small beneficial effect on binding and activation of RXFP4. At RXFP3, the 5-mutation INSL5 showed significantly higher (~5-fold) activation potency than its parent mutant (Fig. 4B and Table 2), suggesting that a Tyr residue at the B2 position had beneficial effects on activation of RXFP3. The measured EC_50_ value of the 5-mutation INSL5 towards RXFP3 reached ~0.5 nM, a value similar to that of INSL5 towards RXFP4 (~0.4 nM). Thus, INSL5 was converted to a strong agonist of RXFP3 *in vitro* through exchange of five exposed B-chain residues of relaxin-3.

In reverse experiments, we replaced B5Tyr of the wild-type R3/I5 and the 4-mutation R3/I5 with the corresponding B2Glu of INSL5. Both R3/I5 mutants showed almost normal binding potencies and slightly lower (2–3-fold) activation potencies towards RXFP4 compared with their parent structures (Figs 3G,H and 4G,H and Table 2). Thus, the N-terminal B5Tyr residue of R3/I5 has only a small contribution to binding and activation of RXFP4. However, [Y(B5)E]R3/I5 showed ~6-fold lower activation potency towards RXFP3 compared with wild-type R3/I5 (Fig. 4C and Table 2), consistent with the fact that introduction of E(B2)Y mutation into the 4-mutation INSL5 led to ~5-fold increase of the activation potency towards RXFP3. The 5-mutation R3/I5 retained high binding potency for RXFP3, but completely lost activation potency towards this receptor due to the presence of the [G(B23)A,G(B24)S] mutation that was an essential determinant for distinguishing RXFP4 from RXFP3.

## Discussion

In the present study, we identified four positions, B2Glu, B9Leu, B17Tyr and a rigid B-chain C-terminus (B20Ala–B21Ser), that were responsible for the high receptor selectivity of INSL5. When these positions were all replaced by the corresponding residues of relaxin-3, the resultant 5-mutation INSL5 acquired high activation potency towards both RXFP3 and RXFP4. When these INSL5 residues were grafted onto the corresponding positions of R3/I5, the resultant R3/I5 mutants all retained high binding and activation potencies towards RXFP4. However, most displayed significantly decreased or even abolished activation potency towards RXFP3, even though they retained high binding potency towards this receptor. Thus, these four positions are the “determinants” underpinning the selectivity of INSL5 for RXFP4 over RXFP3. Among these B-chain determinants, the C-terminal B20Ala–B21Ser and central B9Leu are critical, while B17Tyr and B2Glu are much less important for receptor selectivity.

After the four determinants of INSL5 were all replaced by the corresponding residues of relaxin-3, the resultant 5-mutation INSL5 acquired the highest RXFP3 activation potency. Thus, all these mutations work cooperatively for RXFP3 activation. However, some intermediate mutation combinations had adverse effects on RXFP3 activation, such as the combination of the L(B9)R and Y(B17)F mutations and the combination of the Y(B17)F and [A(B20)G,S(B21)G] mutations. Thus, the high RXFP3 activation potency requires all five mutations in INSL5. The 5-mutation INSL5 also retained normal RXFP4 activation potency, suggesting that the combined mutations were not detrimental to RXFP4 activation. However, some individual mutations had adverse effects on RXFP4 activation when they were present separately, such as the Y(B17)F mutation and the [A(B20)G,S(B21)G] mutation. Their adverse effects could be compensated by the beneficial effects of other mutations, such as the L(B9)R mutation and the E(B2)Y mutation, when all mutations were combined. Thus, the high RXFP4 activation potency of the 5-mutation INSL5 results from a balance of the adverse mutations and the beneficial mutations.

The 5-mutation INSL5 has the highest RXFP3 activation potency reported to date, with an EC_50_ value of ~0.5 nM. However, its activation potency is still ~20-fold lower than that of relaxin-3, the cognate agonist of RXFP3. On the other hand, after these INSL5 determinants were all added to R3/I5, activation potency of the 5-mutation R3/I5 towards RXFP4 was ~13-fold lower than that of INSL5, the cognate agonist of RXFP4. Since the chimeric R3/I5 peptide is as efficient as relaxin-3 for activation of RXFP3, and is more efficient than INSL5 for activation of RXFP4, the relatively lower activities of the 5-mutation INSL5 and the 5-mutation R3/I5 should be attributed to some other B-chain residues that have adverse effects. Therefore, we will identify more residues affecting the receptor selectivity of INSL5 in future studies.

It is thought that a relaxin-3-like peptide is the ancestor of the relaxin family^2^,^43^,^44^. This ancestor peptide might have a broad receptor spectrum, similar to the present relaxin-3 that can activate three of four known relaxin family peptide receptors. During evolution from the ancestor peptide to INSL5, accumulation of mutations in the A-chain likely resulted in loss of RXFP1 activation potency, and accumulation of mutations in the B-chain likely resulted in loss of RXFP3 activation potency, thus INSL5 acquired high receptor specificity. Phylogenetic analysis of the full-length amino acid sequences of relaxin-3 and INSL5 from different vertebrates revealed that INSL5s from mammals occupy one major branch of the phylogenetic tree, while INSL5s from lower vertebrates and relaxin-3s from all vertebrates occupy the other major branch (Fig. 1B). This suggests that INSL5s from the lower vertebrates, such as fishes, birds and reptiles, have a closer relationship to relaxin-3. Alignment of their B-chain sequences (Fig. 1A) indicated that the B23–B24 positions of relaxin-3s are always occupied by two Gly residues in all vertebrates. The corresponding B20–B21 positions of INSL5s are also occupied by two Gly residues in lower vertebrates, including fishes, reptiles and birds; however, these two positions are typically occupied by larger Ala and Ser residues in mammals. Thus, the most important determinant for receptor selectivity of INSL5 (B20Ala–B21Ser) has a late origin and is only present in mammals. For the second important determinant at the B9 position of INSL5 (corresponding to the B12 position of relaxin-3), a similar phenomenon was observed. Thus, the receptor selectivity of INSL5 probably appeared with the origin of mammals during evolution. In the B5 position of relaxin-3, a Tyr residue is highly conserved, but the corresponding B2 position of INSL5s is variable. A highly conserved Phe residue is present at the B20 position of relaxin-3 from all vertebrates, but a highly conserved Tyr residue is present at the corresponding B17 position of INSL5 from all vertebrates. Thus, these two positions of relaxin-3 and INSL5 diverged early during evolution, probably before the origin of vertebrates.

## Methods

### Site-directed mutagenesis of human INSL5 and the chimeric R3/I5

Site-directed mutagenesis of human INSL5 and the chimeric R3/I5 was carried out using the QuikChange methodology. The previously generated expression constructs, pET/INSL5 and pET/R3I5, for overexpression of the single-chain INSL5 and R3/I5 precursors in *E. coli* were used as templates for the initial mutagenesis^38^,^39^. The expression constructs of some INSL5 and R3/I5 mutants were used as templates for subsequent mutagenesis steps. All expected mutations were confirmed by DNA sequencing.

### Preparation of the INSL5 and R3/I5 mutants

The single-chain precursors of the INSL5 and R3/I5 mutants were overexpressed in *E. coli* as inclusion bodies according to our previous procedure^38^,^39^. The precursor of [L(B9)R,Y(B17)F]INSL5 was solubilized using the solubilizing buffer (50 mM phosphate, 0.5 M NaCl, 8 M urea, pH 7.5) without *S*-sulfonation, purified by immobilized metal ion affinity chromatography, and subjected to *in vitro* refolding using a disulfide-reshuffling approach^40^. Other precursors were solubilized in the solubilizing buffer with *S*-sulfonation, purified by immobilized metal ion affinity chromatography, and subjected to *in vitro* refolding using a reduction-oxidation approach^38^,^39^. The refolding mixtures were subjected to HPLC, and the refolded precursors were eluted from a semi-preparative C18 reverse-phase column (Zorbax 300SB-C18, 9.4 × 250 mm, from Agilent Technology, Santa Clara, CA, USA) by an acidic acetonitrile gradient. The folded precursor fraction was manually collected, lyophilized, and subjected to mass spectrometry analysis. Thereafter, the purified folded precursors were sequentially treated with endoproteinase Lys-C and carboxypeptidase B (papaya glutaminyl cyclase was also used for the R3/I5 mutants) according to our previous procedure^38^,^39^, and the resultant two-chain INSL5 and R3/I5 mutants were purified by HPLC using an analytical C18 reverse-phase column (Zorbax 300SB-C18, 4.6 × 250 mm, from Agilent Technology). The fraction of mature mutant eluted by an acidic acetonitrile gradient was manually collected, lyophilized, and subjected to mass spectrometry analysis.

### Circular dichroism measurement

The purified mature INSL5 and R3/I5 mutants were dissolved in 1.0 mM aqueous hydrochloride solution (pH 3.0) and quantified by absorbance at 280 nm, respectively. Their extinction coefficients at 280 nm (ε_280_) were calculated according to the numbers of their Trp and Tyr residues: ε_280nm_ (M^−1^cm^−1^) = 5500 × N_Trp_ + 1490 × N_Tyr_. Thereafter, their final concentrations were adjusted to 20 μM, and circular dichroism measurements were carried out on a Jasco-715 polarimeter at the room temperature. The spectra were scanned from 190 to 250 nm using a quartz cuvette with a 1.0-mm path length.

### Receptor binding assays

The receptor binding assays of the mature INSL5 and R3/I5 mutants were carried out using a NanoLuc-conjugated R3/I5 peptide as tracer and the human embryonic kidney (HEK) 293T cells transiently overexpressing human RXFP3 or human RXFP4 as receptor source. Nonspecific binding was obtained by competition with 1.0 μM of human relaxin-3. Briefly, HEK293T cells were transiently transfected with the expression construct of human RXFP3 or human RXFP4. Next day, the transfected cells were trypsinised, seeded into a 96-well plate, and continuously cultured for 24–36 h to confluence. To start binding assay, the medium was removed and the binding solution (serum-free DMEM medium plus 1% bovine serum albumin) containing 0.5 nM of the NanoLuc-conjugated R3/I5 tracer and varied concentrations of competitor was added (100 μl/well). After incubation at 21 °C for 2 h, the binding solution was removed and the cells were washed twice by ice-cold phosphate-buffered saline (200 μl/well for each wash). Thereafter, the cells were lysed by lysis solution (100 μl/well, from Promega, Madison, WI, USA) and the cell lysate was transferred to a white opaque 96-well plate (50 μl/well). After mixing with the freshly diluted substrate (50 μl/well), bioluminescence was immediately measured on a SpectraMax M5 plate reader (Molecular Devices, Sunnyvale, CA, USA) using the luminescence mode. The measured bioluminescence data were expressed as mean ± SE (*n* = 3) and fitted to sigmoidal curves using the SigmaPlot10.0 software.

### Receptor activation assays

The receptor activation assays of the mature INSL5 and R3/I5 mutants were carried out using a cAMP-response element (CRE)-controlled NanoLuc reporter according to our previous procedure^24^,^25^,^26^. Briefly, HEK293T cells were transiently cotransfected with the expression construct of human RXFP3 or human RXFP4 and the CRE-controlled NanoLuc reporter vector pNL1.2/CRE. Next day, the transfected cells were trypsinised, seeded into a 96-well plate, and continuously cultured for 24–36 h to ~90% confluence. To start activation assay, the medium was removed and the activation solution (serum-free DMEM medium plus 1% bovine serum albumin) containing 1.0 μM of forskolin and varied concentrations of peptide was added (100 μl/well). After being continuously cultured at 37 °C for 4 h, the activation solution was removed and the cells were lysed by lysis solution (100 μl/well, from Promega). The cell lysate was then transferred to a white opaque 96-well plate (50 μl/well), mixed with the freshly diluted substrate (50 μl/well), and bioluminescence was immediately measured on a SpectraMax M5 plate reader (Molecular Devices) using the luminescence mode. The measured bioluminescence data were expressed as mean ± SE (*n* = 3) and fitted to sigmoidal or linear curves using the SigmaPlot10.0 software.

## Additional Information

**How to cite this article**: Hu, M.-J. *et al*. Mechanism for insulin-like peptide 5 distinguishing the homologous relaxin family peptide receptor 3 and 4. *Sci. Rep.* **6**, 29648; doi: 10.1038/srep29648 (2016).

## Figures and Tables

**Table 1:** Molecular masses of the INSL5 and R3/I5 mutants measured by mass spectrometry.

Peptides	Folded Precursors		Mature Peptides	
	Measured	Theoretical	Measured	Theoretical
INSL5	7702.0	7700.8	5632.0	5631.5
[L(B9)R]INSL5	7745.0	7743.9	5675.0	5674.5
[T(B14)A]INSL5	7672.0	7670.8	5603.0	5601.5
[Y(B17)F]INSL5	7686.0	7684.8	5616.0	5615.5
[I(B18)T]INSL5	7691.0	7688.8	5620.0	5619.4
[A(B20)G,S(B21)G]INSL5	7658.0	7656.8	5588.0	5587.4
[L(B9)R,Y(B17)F]INSL5	7731.0	7727.9	5660.0	5658.5
[Y(B17)F,A(B20)G,S(B21)G]INSL5	7643.0	7640.8	5571.0	5571.4
[L(B9)R,A(B20)G,S(B21)G]INSL5	7701.0	7699.8	5631.0	5630.5
[L(B9)R,Y(B17)F,A(B20)G,S(B21)G]INSL5	7686.0	7683.8	5615.0	5614.5
[E(B2)Y,L(B9)R,Y(B17)F,A(B20)G,S(B21)G]INSL5	7718.0	7717.9	5648.0	5648.5
R3/I5	8129.0	8126.3	6169.0	6168.1
[Y(B5)E]R3/I5	8096.0	8092.2	6135.0	6134.0
[R(B12)L]R3/I5	8083.0	8083.3	6126.0	6125.1
[F(B20)Y]R3/I5	8146.0	8142.3	6185.0	6184.1
[G(B23)A,G(B24)S]R3/I5	8174.0	8170.3	6213.0	6212.1
[R(B12)L,F(B20)Y]R3/I5	8100.0	8099.3	6143.0	6141.1
[R(B12)L,G(B23)A,G(B24)S]R3/I5	8130.0	8127.3	6170.0	6169.1
[F(B20)Y,G(B23)A,G(B24)S]R3/I5	8187.0	8186.3	6230.0	6228.1
[R(B12)L,F(B20)Y,G(B23)A,G(B24)S]R3/I5	8145.0	8143.3	6186.0	6185.1
[Y(B5)E,R(B12)L,F(B20)Y,G(B23)A,G(B24)S]R3/I5	8111.0	8109.2	6152.0	6151.0

**Table 2:** Summary of the measured pIC_50_ and pEC_50_ values of the INSL5 and R3/I5 mutants towards RXFP3 and RXFP4.

Peptides	pIC_50_		pEC_50_	
	RXFP3	RXFP4	RXFP3	RXFP4
relaxin-3	8.69 ± 0.03	7.91 ± 0.02	10.61 ± 0.03	9.98 ± 0.04
INSL5	7.26 ± 0.05	7.75 ± 0.02	N.D.	9.36 ± 0.06
R3/I5	8.85 ± 0.02	8.24 ± 0.03	10.44 ± 0.03	10.32 ± 0.03
[L(B9)R]INSL5	7.87 ± 0.04	7.85 ± 0.03	7.21 ± 0.04	9.87 ± 0.04
[T(B14)A]INSL5	7.06 ± 0.03	7.57 ± 0.05	N.D.	9.05 ± 0.04
[Y(B17)F]INSL5	6.77 ± 0.05	7.23 ± 0.03	6.83 ± 0.08	8.40 ± 0.09
[I(B18)T]INSL5	7.07 ± 0.04	7.43 ± 0.05	N.D.	9.31 ± 0.08
[A(B20)G,S(B21)G]INSL5	6.01 ± 0.06	6.39 ± 0.05	6.90 ± 0.04	8.59 ± 0.05
[L(B9)R,Y(B17)F]INSL5	8.17 ± 0.04	7.33 ± 0.03	<6	9.20 ± 0.03
[Y(B17)F,A(B20)G,S(B21)G]INSL5	<6	6.30 ± 0.05	<6	8.00 ± 0.07
[L(B9)R,A(B20)G,S(B21)G]INSL5	6.75 ± 0.05	6.99 ± 0.08	8.12 ± 0.04	9.23 ± 0.04
[L(B9)R,Y(B17)F,A(B20)G,S(B21)G]INSL5	7.37 ± 0.03	7.54 ± 0.06	8.61 ± 0.04	9.46 ± 0.06
[E(B2)Y,L(B9)R,Y(B17)F,A(B20)G,S(B21)G]INSL5	7.54 ± 0.03	7.70 ± 0.03	9.27 ± 0.04	9.78 ± 0.03
[Y(B5)E]R3/I5	8.52 ± 0.03	8.22 ± 0.04	9.64 ± 0.03	10.08 ± 0.05
[R(B12)L]R3/I5	6.79 ± 0.02	7.79 ± 0.03	7.23 ± 0.04	9.45 ± 0.04
[F(B20)Y]R3/I5	8.66 ± 0.04	7.80 ± 0.05	10.01 ± 0.03	9.14 ± 0.06
[G(B23)A,G(B24)S]R3/I5	8.61 ± 0.03	8.06 ± 0.04	N.D.	8.44 ± 0.04
[R(B12)L,F(B20)Y]R3/I5	7.13 ± 0.04	7.77 ± 0.04	7.13 ± 0.04	9.00 ± 0.06
[R(B12)L,G(B23)A,G(B24)S]R3/I5	8.38 ± 0.03	7.83 ± 0.04	N.D.	8.16 ± 0.04
[F(B20)Y,G(B23)A,G(B24)S]R3/I5	8.45 ± 0.04	7.91 ± 0.02	N.D.	9.02 ± 0.05
[R(B12)L,F(B20)Y,G(B23)A,G(B24)S]R3/I5	8.40 ± 0.04	7.75 ± 0.05	N.D.	8.71 ± 0.06
[Y(B5)E,R(B12)L,F(B20)Y,G(B23)A,G(B24)S]R3/I5	8.11 ± 0.02	7.61 ± 0.05	N.D.	8.24 ± 0.05

(N.D., not detectable).
